# Sensitivity and specificity of automated analysis of single-field non-mydriatic fundus photographs by Bosch DR Algorithm—Comparison with mydriatic fundus photography (ETDRS) for screening in undiagnosed diabetic retinopathy

**DOI:** 10.1371/journal.pone.0189854

**Published:** 2017-12-27

**Authors:** Pritam Bawankar, Nita Shanbhag, S. Smitha K., Bodhraj Dhawan, Aratee Palsule, Devesh Kumar, Shailja Chandel, Suneet Sood

**Affiliations:** 1 Sri Sankaradeva Nethralaya, Guwahati, India; 2 Department of Ophthalmology, Dr. D.Y Patil Hospital & Research Centre, Mumbai, India; 3 KLES Dr. Prabhakar Kore Hospital & Research Centre, Belgavi, Karnataka, India; 4 NKP Salve Institute of Medical Sciences and Research Center, Nagpur, Maharashtra, India; 5 Deenanath Mangeshkar Hospital, Pune, Maharashtra, India; 6 Think-i, Noida, Uttar Pradesh, India; 7 Jeffrey Cheah School of Medicine and Health Sciences, Monash University Malaysia, Johor Bahru, Malaysia; Soochow University Medical College, CHINA

## Abstract

Diabetic retinopathy (DR) is a leading cause of blindness among working-age adults. Early diagnosis through effective screening programs is likely to improve vision outcomes. The ETDRS seven-standard-field 35-mm stereoscopic color retinal imaging (ETDRS) of the dilated eye is elaborate and requires mydriasis, and is unsuitable for screening. We evaluated an image analysis application for the automated diagnosis of DR from non-mydriatic single-field images. Patients suffering from diabetes for at least 5 years were included if they were 18 years or older. Patients already diagnosed with DR were excluded. Physiologic mydriasis was achieved by placing the subjects in a dark room. Images were captured using a Bosch Mobile Eye Care fundus camera. The images were analyzed by the Retinal Imaging Bosch DR Algorithm for the diagnosis of DR. All subjects also subsequently underwent pharmacological mydriasis and ETDRS imaging. Non-mydriatic and mydriatic images were read by ophthalmologists. The ETDRS readings were used as the gold standard for calculating the sensitivity and specificity for the software. 564 consecutive subjects (1128 eyes) were recruited from six centers in India. Each subject was evaluated at a single outpatient visit. Forty-four of 1128 images (3.9%) could not be read by the algorithm, and were categorized as inconclusive. In four subjects, neither eye provided an acceptable image: these four subjects were excluded from the analysis. This left 560 subjects for analysis (1084 eyes). The algorithm correctly diagnosed 531 of 560 cases. The sensitivity, specificity, and positive and negative predictive values were 91%, 97%, 94%, and 95% respectively. The Bosch DR Algorithm shows favorable sensitivity and specificity in diagnosing DR from non-mydriatic images, and can greatly simplify screening for DR. This also has major implications for telemedicine in the use of screening for retinopathy in patients with diabetes mellitus.

## Introduction

Diabetic retinopathy (DR) in time affects nearly all individuals with type I,[1] and most patients with type II diabetes melllitus.[2] DR may progress to blindness; in fact, DR is the leading cause of new blindness among working-age adults[3]. About 4% of persons with early-onset diabetes are blind, and nearly all blindness in this age group is related to the complications of DR.[3] About 2–3% of late-onset diabetics are blind.[4]–in this group about a third of blindness is caused by diabetes.[3]

The risk of DR can be reduced by careful control of blood sugar levels and blood pressure.[5, 6] Early diagnosis of DR through effective screening programs, should, therefore, be expected to improve vision outcomes. DR has a long latent phase, and screening and timely intervention for DR has been shown to be cost-effective when compared with the disability loss of blindness.[7, 8] Indeed, Ferris[9] estimated that blindness would be reduced ten-fold with appropriate early intervention.

Fundus photography is a simple and cost-effective method of making a diagnosis in suspected retinopathy. One of the major advantages is that the photograph can be examined by others, at various locations, much as an X-ray can.[10] The first photographs of the retina were published in 1886, but commercial fundus cameras appeared only forty years later.[11]

The gold standard for grading DR is the expert interpretation of the ETDRS seven-standard-field 35-mm stereoscopic color retinal image (ETDRS) of the dilated eye.[12, 13] Traditional fundus cameras provide excellent pictures, but are typically large, bulky, difficult to use. They are also expensive, and require time-consuming and uncomfortable mydriasis, and are therefore clearly not designed for screening. Since screening for DR is often inadequate, there is a need for easily-available, inexpensive methods for diagnosing the condition.[14] Over the years, progress has been directed towards non-mydriatic photography, simplifying cameras, diagnosis by general practitioners, and automated diagnosis. Non-mydriatic image acquisition methods take less than half the time[15] and, when interpreted by ophthalmologists, show good correlation with the gold standard.[15, 16] Telemedicine has also helped overcome the shortage of qualified ophthalmologists.[17]^,^[18]

Despite these endeavors, screening still may be inadequate.[19, 20] A screening method that does not require trained persons would be expected to significantly improve availability and reduce costs.[21]

We have been working on a medical image analysis application for ophthalmology professionals. This application can capture retinal images and transfer them to a computer (and to a cloud database), and can classify images as healthy, inconclusive, or DR affected. In the last year, we have prospectively evaluated this system for the diagnosis of DR. The objective of the paper is to present the sensitivity, specificity, positive predictive value (PPV), and negative predictive value (NPV) of this instrument, with respect to 7-field ETDRS imaging.

## Methods

The study was a prospective, open-label trial. Non-mydriatic images were captured using the Bosch camera and were evaluated by the Bosch DR Algorithm software for the diagnosis of DR. The results were compared with those obtained by 7-field ETDRS images in the same subjects.

### Patients

The study was conducted on patients recruited at one of 6 centers in India (Table 1).

**Table 1:** Participating centers in India.

1	Padmashree Dr. DY Patil Medical College Hospital and Research Centre, Mumbai, Maharashtra.
2	JN Medical College, KLE University, Belgavi, Karnataka.
3	Dr. Virendra Laser, Phaco Surgery Centre Pvt. Ltd., Jaipur, Rajasthan (SEAROC Ethics Committee, Jaipur, Rajasthan).
4	Sri Sankaradeva Nethralaya, Guwahati, Assam.
5	Deenanath Mangeshkar Hospital and Research Centre, Pune, Maharashtra.
6	NKP Salve Institute of Medical Sciences and Lata Mangeshkar Hospital, Nagpur, Maharashtra.

The study was approved by the ethics committees of each of the participating hospitals and medical centers. For the center at Jaipur, the approval was provided by the ethics committee of the SEAROC Cancer Center, Jaipur. The study was registered on the Clinical Trials Registry–India (CTRI), with the registration number CTRI/2017/01/007709 (the trial protocol can be viewed at the following url: http://ctri.nic.in/Clinicaltrials/showallp.php?mid1=16105&EncHid=&userName=thinki). Informed written consent was taken, and Good Clinical Practice norms were followed at all times.

Male and female patients suffering from type I or type II diabetes mellitus for at least 5 years were included if they were 18 years or older. We excluded patients who were already diagnosed to have DR, and those who had associated intraocular disorders (Table 2).

**Table 2:** Exclusion criteria.

Inability or unwillingness to provide an informed consent
History of known retinal disease
History of intraocular surgery (other than cataract surgery), or of ocular laser or injection treatment for any retinal disease
Extremely small pupil that affected image capture, or an opacity or other condition in either eye that precluded good bilateral retinal photography
Conjunctivitis, red eye, or any other inflammatory condition with photophobia
Gestational diabetes mellitus
Inability or unwillingness to provide an informed consent

### Diagnosis of DR

The algorithm diagnosed the eyes as “healthy”, “DR affected” or “inconclusive”.

The investigators who read the images diagnosed the eyes as “DR present” or “DR absent”. They used the American Academy of Ophthalmology guidelines[22] as the standard for making the diagnosis. Any findings falling under the nonproliferative DR or proliferative DR category of ETDRS[12] were categorized as “DR present”.

### Fundus image collection

Once subjects were enrolled, a medical history was obtained and a clinical examination carried out. The subjects were placed in a dark room for at least two minutes to achieve physiological mydriasis before they underwent retinal imaging.

A single external fundus image of each eye was acquired using the Bosch nonmydriatic fundus camera. This camera is a physical hand-held device that offers both non-mydriatic and mydriatic modes for faster and accurate detection. We captured non-mydriatic single-field color fundus images with effective spatial resolutions equal or more than 2.6 MP. Images were uploaded to the cloud using the telemedicine software, Medibilder Lite. There were first pre-processed by cropping (to get approximately square images), re-scaled to a lower resolution of 512*512, and then normalized and transferred to the Bosch algorithm for DR evaluation. The results from the algorithm were documented. Investigators were kept blinded from the results.

After the nonmydriatic images were taken, the patients underwent 7-Standard Field stereoscopic Digital Colour Fundus (EDTRS) imaging[12] after mydriasis with tropicamide. These images were evaluated by the investigators.

### Deep learning algorithm for DR classification

**Fig 1:**
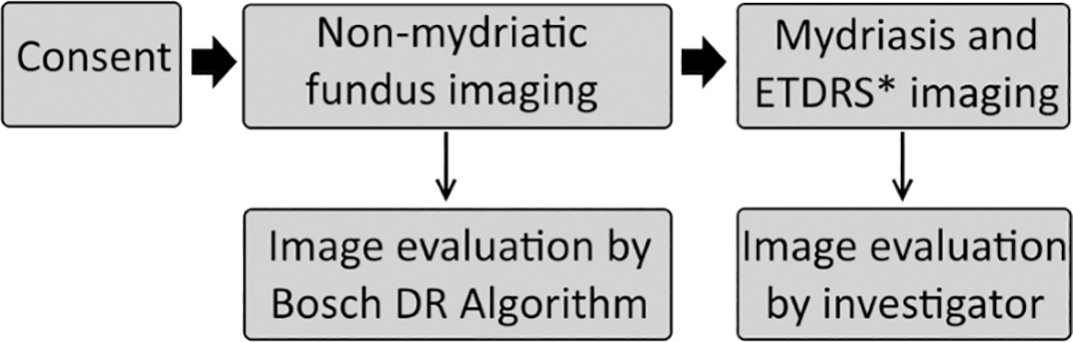
Process flow of subjects. *ETDRS: ETDRS seven-standard-field 35-mm stereoscopic color retinal imaging.

The fundus images captured from the patients were analyzed by the Bosch DR Algorithm, which performs classification. The image data acquired was processed and the output generated using MediBilder as a user-interface. Medibilder is a DICOM compliant client software for picture archiving and communication systems, capable of image operations such as marking, annotation, and layering. (DICOM is an acronym for Digital Imaging and Communications in Medicine standard for handling, storing, printing, and transmitting information in medical imaging.) This algorithm classifies each fundus image as DR affected, healthy, or inconclusive. The data were recorded into electronic case record forms (CRFs). Fig 1 describes the process flow.

The Bosch DR Algorithm uses deep convolutional neural network to automatically detect whether a fundus image has DR. Deep learning is an artificial intelligence method in which the input image passes through many types of filters in order to automatically extract the best features.[23] The computational models involve numerous convolutional layers, and the automatically generated features have markedly improved the state-of-the-art in visual object recognition.^[^^24^^]^ Our deep learning architecture configuration consists of 13 convolutional layers. The optimal number of layers was based on experimental analysis, and we found that increasing the layers did not raise the performance significantly. We have used cross-entropy as the cost function to be minimized. The output layer consists of a single output neuron (for specifying the result of binary classification).

The Bosch algorithm differs from one developed by the Google team[25] in two main ways.

One, the Google network uses a more complex architecture that consists of 11 blocks, and, totally, there are more than 80 convolutional layers. They had used an ensemble of 10 such networks. We have used a different scheme of a single network, which consists of 13 convolutional layers, and yet it provides sufficient accuracy.

Two, in our network, to avoid the problem of vanishing gradients (wherein the gradient update tends to zero), we adopted the technique of adding random noise to the gradients during optimization, as described by Neelakantan et al.[26] This approach also helps to avoid overfitting, and results in lower training loss. On the other hand, this addition of random noise was not found in Google’s paper.

**Inconclusive cases**: The proportion of inconclusive cases is constrained to be around 10%-20% of the test data by optimizing the thresholds (which is ensured during training).

**Training database**: For the training phase, we have used a large dataset of nearly 80,000 images. These consist of challenging cases from (i) Open-source data EyePACS-1, that comprises mydriatic and non-mydriatic fundus images, and (ii) About 5000 Bosch Eye Camera images collected from various camps across India, verified by 3 ophthalmologists. The training set was derived from completely different patients, sites, and operators, when compared to the testing images of the actual study. This ensures external validity of the algorithm, as the test set is different.

### Sample size calculations and statistics

We calculated the sample size as follows:

N = (Z^2^ * Sn * (1-Sn))/ (L^2^ * prevalence), where Z was 1.96 for alpha = 0.05, Sn = sensitivity, set at 0.9 from previous studies, L = margin of error, set at 0.05, and prevalence (of DR in diabetes) set at 0.25 from earlier studies.[27–29] This provided a requirement of 533 subjects, which we rounded off to 550.

Data analysis was carried out using SPSS® version 22. The sensitivity, specificity, PPV, and NPV were calculated for the algorithm using the investigators’ diagnoses on the 7 field ETDRS images as the gold standard. We excluded the cases classified as inconclusive by the algorithm.

## Results

**Fig 2:**
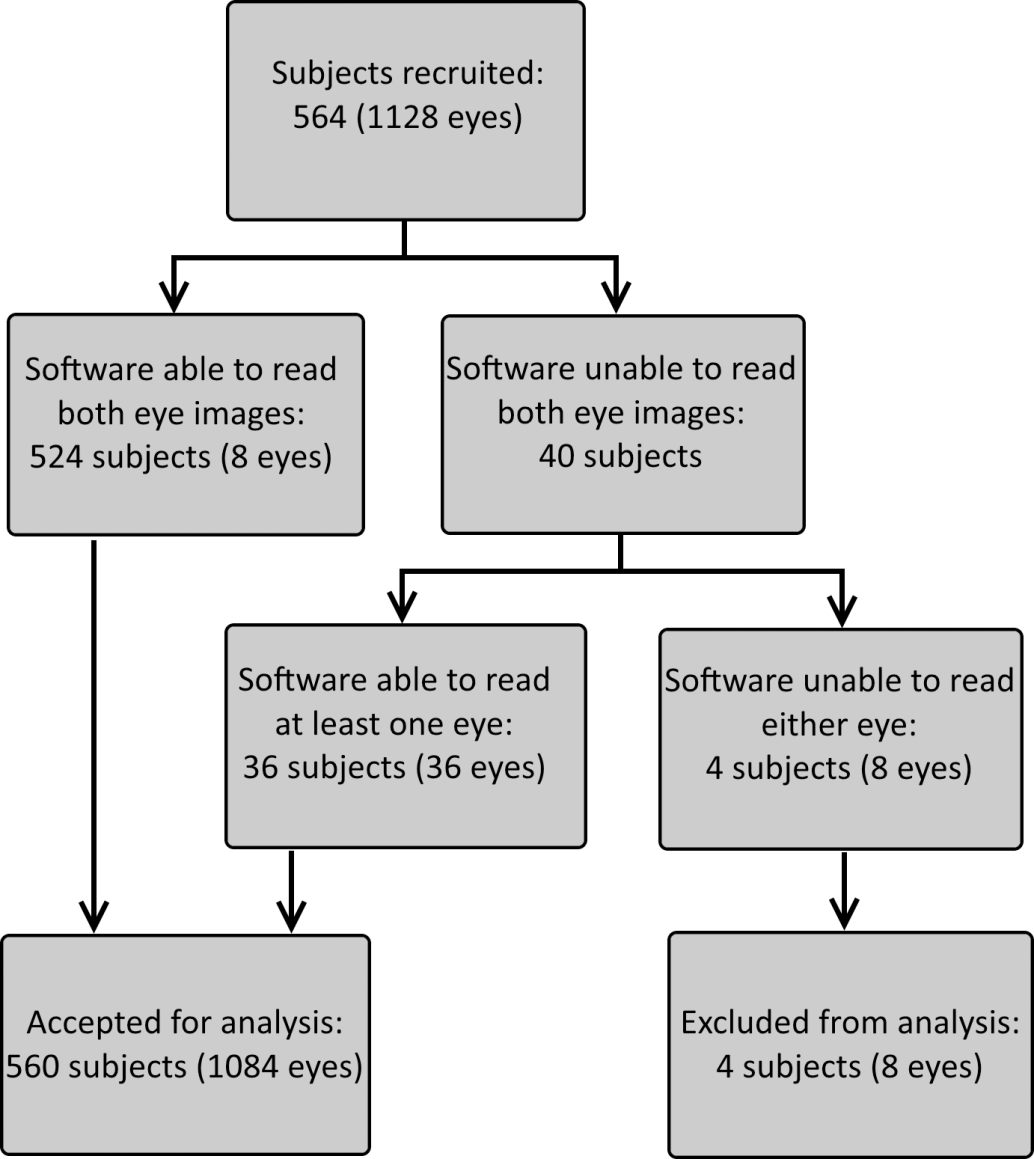
Flow chart showing recruitment, inclusion, and exclusion of subjects.

Subjects were recruited between October 2016 and January 2017. The study included 564 subjects, each of whom were evaluated at a single outpatient visit. Of the 1128 eyes studied, 44 images (3.9%) were categorized inconclusive by the algorithm. Eight of these were from the same four subjects, who were excluded because the algorithm categorized both eyes as inconclusive. This left 1084 eyes, from 560 subjects, for analysis (Fig 2). The subjects included 351 males and 209 females, with ages ranging from 20 to 85 years (median 58). They all had diabetes for at least five years.

### Bosch DR Algorithm

The algorithm correctly diagnosed 531 of 560 cases (Table 3). The sensitivity, specificity, PPV, and NPV were 91.18%, 96.9%, 94.4%, and 95.0% respectively.

**Table 3:** Sensitivity and specificity of the Bosch Dr Algorithm.

Bosch DR Algorithm	7-field ETDRS imaging	Result	Cases (total: 560)
Positive	Positive	True positive	186
Negative	Positive	False negative	18
Negative	Negative	True negative	345
Positive	Negative	False positive	11

Sensitivity 91.18% (86.41–94.69), Specificity 96.91% (94.54–98.45), PPV 94.4% (90.42–96.81), NPV 95.0% (92.5–96.75), positive likelihood ratio value 29.51 (16.47; 52.88), negative likelihood ratio value 0.09 (0.06; 0.14). Figures in parentheses represent 95% confidence limits.

## Discussion

Non-mydriatic fundus photography became available in the 1980s.[30] It was, naturally, well-tolerated by patients, but the images sometimes lacked clarity, with 5–25% of pictures being unusable. Early studies showed that though the specificity rates were high, sensitivity rates for the diagnosis of DR were low, missing nearly half of cases.[14] Improvement in cameras was driven by the frequent occurrence of unusable images. An excellent review was published by Panwar et al,[11] detailing the technical specifications of fundus cameras for mydriatic and non-mydriatic photography. More recently, cameras have become smaller: indeed, some mobile phones are able to take pictures without the need for mydriasis.[11]

### Image quality

In 1084 of 1128 cases the images were of acceptable quality for evaluation (96.1%). This compares favorably with the rates reported in earlier publications, where about 70–95% of non-mydriatic photographs were acceptable.[14, 31–34]

Our software has advantages over previously described work, for example that published recently by Google’s Gulshan and coworkers.[25] Unlike the California-based team, which used an ensemble of 10 networks, we have used a much simpler network and yet obtained comparable performance. Fewer layers require lesser memory and processing power. An additional novelty of our architecture is that we have added noise to the gradients (during optimization). This modification, which is included in the training model, helps us in providing robust results even for lower quality images, with variations such as color and visibility of lesion-affected regions.

The Kaggle diabetic retinopathy challenge[35] released a huge publicly available dataset, wherein several deep learning-based approaches wee proposed to classify different grades of retinopathy. In our work, we only focus on referral/no referral classification for DR versus healthy images. The diagnostic accuracy of computer detection of DR was reported by Abramoff and coworkers,[36] who also provided a detailed clinical study.[37] A detailed survey of DR detection algorithms from fundus images was presented in a review by Mookiah et al,[38] but these dealt with lesion detection-based approaches and considered only much smaller public databases such as DIARETDB1.[39]

### Sensitivity and specificity

Non-mydriatic photography is convenient, but sensitivity and specificity have always been a concern.

The best results are obtained when an ophthalmologist reads the images. Reports show sensitivity rates varying from 78%[40] to 96%[41]. Specificity is typically higher, ranging from 86%[40] to 98%[41]. In a small study of 55 subjects, Vujosevic et al[42] reported a sensitivity of 99%, and a specificity of 100%.

Recognizing that an ophthalmologist is not always available in rural areas,[43] healthcare personnel have used trained general practitioners to interpret the images. Castro and coworkers[44] reported sensitivity rates of 67% for non-mydriatic examination, but more recent reports indicate that the sensitivity for diagnosis is between 83–97% when carried out by general practitioners, with most publications reporting rates close to 95%.[45, 46]

Over the years, progress has also been made in identifying retinal lesions using algorithms to diagnose changes in digitized images.[10, 47–49] Abramoff and coworkers[50] evaluated an algorithm-based system for automated detection of DR in retinal photographs. They achieved a sensitivity of 0.84, but a low specificity, and concluded that automated detection showed promise, even if it was not immediately suitable for clinical use. Recently, Besenczi et al,[10] Schuster et al,[51] and Rahim et al[52] have described the components of automated detection programs. The software is able to localize the optic disc and the macula, and distinguish the arteries from the veins.[53] It can detect retinal lesions such as microaneurysms, exudates, and others. Changes such as papilledema,[54, 55] hemorrhages,[56] neovascularization,[57] and exudates[58] can be identified, typically with sensitivity rates approaching 80% and overall accuracy rates greater than 90%.

Putting these programs together, attempts have been made for the automated diagnosis DR from images. Tufail et al[59] stated that the sensitivity of EyeArt, their automated DR image assessment systems (ARIAS) was 94.7%, when compared to manual graders. Unfortunately their program setting appeared to achieve this high sensitivity at the cost of specificity, and the false-positive rate approached 80%. It was also not clear from their papers[59, 60] whether the images were consistently non-mydriatic. Bhaskaranand et al[61] reported similar, but slightly less accurate, results, also using EyeArt. Using mydriasis, Hansen et al,[62] in Kenya, used the Iowa Detection Program and set an optimum balance between sensitivity and specificity, achieving rates of 91% and 70% respectively.

There are few studies that have specifically evaluated the role of automated image analysis of non-mydriatic photography using mydriatic photographs as a standard. Hansen et al,[63] from Denmark, reported a sensitivity of 90% and a specificity of 85.7% in a small group of 83 subjects, and recommended that automated systems could be used effectively for screening. In the present study, using the ophthalmologist’s diagnosis from 7-field ETDRS images as the gold standard, the Bosch DR Algorithm achieved sensitivity, specificity, PPV, and NPV rates of 91%, 96%, 94%, and 95% respectively. The British Diabetic Association recommends that screening programs for DR should reach sensitivity and specificity levels of 80% of higher,[64] and the Bosch DR Algorithm comfortably surpasses this requirement. Our results compare favorably with those obtained by other workers, and we consider that this algorithm can be an effective instrument for screening for DR.

### Telemedicine benefits

Despite well-established guidelines for screening, patients often present with DR-related blindness. The cause is probably inadequate screening,[65] and telemedicine with remote interpretation may be an important strategy for tackling this problem. Fortunately, DR is a condition that lends itself easily to the benefits of telemedicine.[66, 67] Studies have repeatedly confirmed the importance of telemedicine as an important screening tool in different parts of the world,[68–70] and it is more effective than traditional surveillance.[71] In a country like India, where a third of the rural population of the needs to travel over 30 kilometers for access to basic medical treatment,[72] “tele-ophthalmology” can be particularly valuable.[73] Non-mydriatic imaging is highly acceptable to patients.[74] The main problems are those of image inconsistency,[75] which highlights the importance of any system that can provide quality images with a low rate of unreadable pictures.

### Strengths and weaknesses

This study compares non-mydriatic imaging with an acceptable gold standard. We consider that our other strength is in the large number of subjects, which should provide valid measures of accuracy using this technology.

In our study, a subject was considered for analysis if even one eye had yielded an adequately clear image. In some of the eyes diagnosed as normal, the other eye may well have had evidence of early DR. Further, while the study notes the findings of DR, it would be useful to know how accurate this software is for individual lesions, such as exudates, microaneurysms, and macular edema.
